# Evaluation of the Placement of Mobile Fruit and Vegetable Vendors to Alleviate Food Deserts in New York City

**DOI:** 10.5888/pcd11.140086

**Published:** 2014-09-11

**Authors:** Kathleen Y. Li, Ellen K. Cromley, Ashley M. Fox, Carol R. Horowitz

**Affiliations:** Author Affiliations: Ellen K. Cromley, University of Connecticut School of Medicine, Storrs, Connecticut; Ashley M. Fox, Carol R. Horowitz, Icahn School of Medicine at Mount Sinai, New York, New York.

## Abstract

**Introduction:**

In 2008, the New York City (NYC) health department licensed special mobile produce vendors (Green Carts) to increase access to fruits and vegetables in neighborhoods with the lowest reported fruit and vegetable consumption and the highest obesity rates. Because economic incentives may push vendors to locate in more trafficked, less produce-deprived areas, we examined characteristics of areas with and without Green Carts to explore whether Carts are positioned to reach the intended populations.

**Methods:**

Using ArcGIS software, we mapped known NYC Green Cart locations noted through 2013 and generated a list of potential (candidate) sites where Carts could have located. We compared the food environment (via categorizing “healthy” or “unhealthy” food stores using federal classification codes corroborated by online storefront images) and other factors that might explain Cart location (eg, demographic, business, neighborhood characteristics) near actual and candidate sites descriptively and inferentially.

**Results:**

Seven percent of Green Carts (n = 265) were in food deserts (no healthy stores within one-quarter mile) compared with 36% of candidate sites (n = 644, *P* < .001). Most Carts (78%) were near 2 or more healthy stores. Green Carts had nearly 60 times the odds of locating near subway stops (*P* < .001), were closer to large employers (odds ratio [OR], 6.4; *P* < .001), other food stores (OR, 14.1; *P* < .001), and in more populous tracts (OR, 2.9, *P* <.01) compared with candidate sites.

**Conclusion:**

Green Carts were rarely in food deserts and usually had multiple healthy stores nearby, suggesting that Carts may not be serving the neediest neighborhoods. Exploration of Carts’ benefits in non–food desert areas is needed, but incentivizing vendors to locate in still-deprived places may increase program impact.

## Introduction

Inadequate access to fresh fruits and vegetables, particularly in low-income areas, is believed to be a cause of obesity and obesity-related disparities in health (1,2). The term “food desert” references areas where residents lack adequate access to fresh fruits and vegetables. No universal definition exists, but the US Department of Agriculture defines food deserts in urban areas as communities more than 1 mile from the nearest supermarket (3). Food deserts may be one explanation for disparities in obesity rates, although studies of their impact on obesity and diet reached mixed conclusions (4–6). Because of concerns about obesity and obesity disparities, local public health departments have begun exploring policies to increase consumption of fresh produce in low-income, food desert areas. However, it is challenging for governments to choose the “best” solution when little evidence supports one policy over another (7). The purpose of this article is to evaluate one such approach: the New York City (NYC) Green Carts program.

In 2008, the NYC Department of Health and Mental Hygiene (DOHMH) established 1,000 permits for Green Carts, privately owned and operated mobile fruit and vegetable vendors, to increase fresh produce availability in neighborhoods with the lowest reported rates of fruit and vegetable consumption, specifically East and Central Harlem, South Bronx, North and Central Brooklyn, and portions of Queens and Staten Island (8). Although Green Carts may locate anywhere within these designated areas, we hypothesized that, for economic reasons, Cart vendors may choose to situate in busy commercial areas that already have fruits and vegetables rather than in food deserts. One study demonstrated that Carts cluster near highly trafficked areas in the Bronx (9), but we found no studies assessing whether the program is fulfilling its stated goal of helping New Yorkers “buy fresh fruit and vegetables close to home” (10). Therefore, we aimed to assess to what degree Green Carts improve food access in the program’s target neighborhoods and to determine how other demographic, business, and neighborhood factors influence Cart placement. A secondary aim was to determine whether economically viable Green Cart sites in food deserts exist, which could increase the program’s reach.

## Methods

### Data sources and sample

The NYC DOHMH, which regulates and inspects the Carts, provided a list of intersections where Carts were located (updated periodically during 2008–2013) and those capable of accepting Electronic Benefits Transfer (EBT) for the Supplemental Nutrition Assistance Program (SNAP) (food stamps). We obtained Green Cart boundaries (areas of the city where Carts are allowed) from the NYC DOHMH website (8). Because Carts can be patronized by people living outside but near designated Cart zones, we extended the study area to include a half-mile buffer beyond Cart boundaries. All distances were measured along the street network from a downloadable database (NYC Bytes of the Big Apple street centerline data, 2013 [http://www.nyc.gov/html/dcp/html/bytes/applbyte.shtml#lion]). We also included a half-mile buffer around any Carts outside the designated Cart boundaries (“outliers”). We excluded Staten Island because it had no active Cart permits. All mapping was done using ArcGIS software (Esri). Demographic and neighborhood characteristics of census tracts partially or entirely within the study area were analyzed.

### Candidate Green Cart locations

Rather than only examining neighborhoods with Green Carts, we generated comparison “candidate” sites in census tracts where Carts could have located but did not. For census tracts strictly within Cart boundaries that lacked Green Carts, we designated the intersection closest to the geographic center of the tract as a candidate site.

#### Food deserts and food environment

To assess the food environment around Green Carts, we first identified “healthy” and “unhealthy” food stores inside the study area using InfoUSA North American Industry Classification System (NAICS) codes (ArcInfo Business Analyst extension 2012 [Esri]) for supermarkets, fruit and vegetable specialty stores, warehouse clubs, fast food restaurants, convenience stores, and other grocery stores based on codes the Centers for Disease Control and Prevention (CDC) includes in its census tract–level food environment analysis. CDC also classifies businesses as healthy or unhealthy on the basis of its number of employees (11), but this method often results in store misclassification (12–14).

To more accurately measure the food environment, we created an initial list of food retail establishments within the study area based on the CDC’s NAICS codes. Next, we crosschecked each listed store using Google Maps Street View images (generally taken from 2011 and 2012) and available store websites to verify store presence and to identify smaller stores that also carry fresh produce. We used Web-based store locator functions to identify additional supermarkets not in the InfoUSA database. We defined “healthy” stores as chain supermarkets, fruit and vegetable specialty stores, warehouse clubs, or small or medium stores with evidence of selling fruits and vegetables based on Google Street View.

Stores coded as convenience stores, fast food restaurants, and small or medium storefronts without evidence of selling fruits and vegetables (and which predominantly sold beer, soda, sandwiches, and cigarettes) were classified as “unhealthy.” The code for fast food restaurants changed between 2007 (used in the most recent CDC food environment reports) and 2012 (the most current database). Thus, we identified fast food restaurants by common names such as McDonald’s and Popeye’s, as well as by names containing the words “fried chicken,” “burger,” “burrito,” “taco,” “pizza,” “take out,” “to-go,” and “express.” Miscoded establishments (wholesalers, stores not selling food, and full-service restaurants), duplicate addresses, residential addresses, and unidentifiable addresses were excluded from analysis.

We validated our food environment measure via store audits in 2 zip codes, 1 in the Bronx and 1 in Manhattan, representing approximately 5% of stores we identified. We canvassed these neighborhoods on foot in May 2014 to verify the accuracy of our methods in ascertaining both store count and healthy or unhealthy classification. Stores selling 4 or more types of fruits and vegetables were considered healthy. Our study methods classified healthy or unhealthy stores correctly more than 95% of the time, and we found no additional healthy stores excluded from our analysis that would lead to concerns about misclassifying food deserts, though some unhealthy stores were undercounted by our method.

After classifying stores as healthy or unhealthy, we categorized the food environment within a quarter-mile street network distance from Green Carts and candidate sites as being food deserts (0 healthy stores), food swamps (≤1 in 5 healthy stores), or healthy areas (>1 in 5 healthy stores) based on the proportion of healthy stores to the total (healthy and unhealthy). These methods are similar to CDC’s calculation of a modified retail food environment index (11). Food deserts/swamps have been defined variably, including within administrative areas (eg, census tracts or zip codes) or at varying distances from a residence or other point, ranging from a few hundred meters to 3 miles (15). Given the high population and retail density and the reliance on public transportation and walking in the city, and on the basis of a prior study of the food environment in NYC that defined accessibility as a quarter-mile (about 5 minutes walking distance) (16), we similarly conservatively defined a food desert as lacking any healthy food stores within a quarter-mile of a given location, although we also evaluated each variable at the half-mile distance.

#### Census tract and neighborhood characteristics

We examined other neighborhood characteristics, such as distance to large businesses and subway stops and census tract–level demographic information, around Carts and candidate sites to better understand why Green Cart vendors chose to locate in certain areas. We hypothesized that because Cart vendors may locate anywhere within the designated boundaries, economic incentives would drive them to locate in areas maximizing their customer base, such as near large employers, subway stops and higher income areas with customers that might have a higher demand for fruits and vegetables.

Distance to large employers and subway stops was calculated for each actual and candidate Green Cart location. We gathered large employer data from ArcInfo Business Analyst extension 2012, defining small businesses as having fewer than 500 employees (17) and used the Metropolitan Transportation Authority downloadable geographic information systems database of subway stops for 2013 (http://web.mta.info/developers/sbwy_entrance.html). Tract demographic information including population size, mean household income, proportion of the population below the federal poverty level, and racial/ethnic composition of the tract was obtained from the American Community Survey (http://factfinder2.census.gov/faces/nav/jsf/pages/searchresults.xhtml?refresh=t). To account for changing demographic trends, we used 5-year sample estimates of data on demographic variables obtained from the American Community Survey for 2008 through 2012.

### Analysis

We compared neighborhood characteristics of all known Green Carts to candidate sites to assess whether Carts were serving areas with very limited access to fresh produce, as the program intended. We used independent sample *t* tests and χ^2^ tests of association to compare census tract–level demographic information, the business environment (number of large employers), and the food environment for actual and candidate Cart sites using SAS 9.3 (SAS Institute, Inc). We conducted multivariable logistic regression analyses to determine how the food environment was associated with Cart presence and how demographic and other neighborhood characteristics influenced Green Cart placement. Because we expected that vendors may be likely to locate in more commercial areas regardless of food environment, we also included the total number of food stores within a quarter-mile as a predictor in 1 regression model. However, because sites with 0 food stores nearby are by definition also food deserts, food environment type and density were not both included in any model. We also examined neighborhood differences between Green Carts inside and outside the designated boundaries and between Green Carts that accept and do not accept EBT.

## Results

The NYC Department of Health provided a list of 265 active Green Cart locations out of 494 issued permits as of August 2013 (8), which mapped to 154 unique census tracts, including 22 “outlier” Carts outside the designated boundaries (Figure). A minority of Carts (n = 43) accepted EBT. We generated 644 candidate sites from the remaining census tracts within Cart boundaries. We identified 979 healthy and 1,579 unhealthy stores within the study area. Overall, we counted more than 75% more healthy food stores than were found using CDC criteria based on number of employees only.

**Figure:**
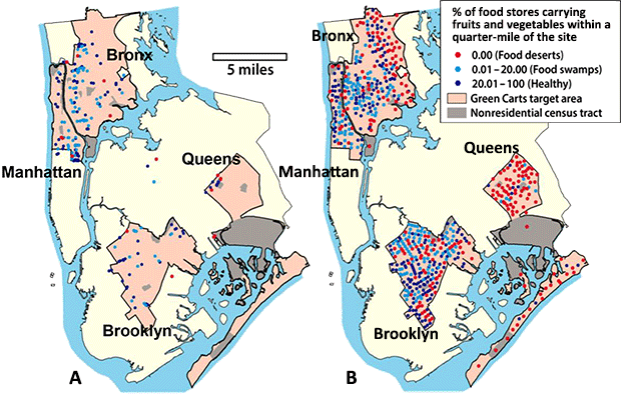
Green Cart (panel A) and candidate locations (panel B) shown by food environment within a quarter-mile street network distance, New York City. Cart locations were obtained from New York City Department of Health and Mental Hygiene, updated through 2013. Food store locations identified with ArcInfo Business Analyst 2012 (Esri) were verified via Google Street View.

Compared with candidate sites, Green Carts were much more likely to be near healthy stores (Table 1). Seven percent of 265 Green Carts were in food deserts, and most (78%) were near 2 or more existing healthy stores. In comparison, 36% of 644 candidate sites were in food deserts (*P* < .001), and 42% had 2 or more healthy stores nearby. Substantial portions of the Bronx and Queens had few Green Carts (Figure) yet had many candidate sites in food deserts where Carts could be located. Many Carts (41%) were in food swamps, but over half (51%) were in healthy areas, while 29% of candidate sites were in food swamps and 35% were in healthy areas. Of all 108 sites with no stores nearby, only 4 were actual Green Cart locations. In contrast, Green Carts were in locations that had many (16 or more) food stores nearby (130 vs 91 candidate sites).

**Table 1:** Neighborhood Characteristics of Green Cart and Candidate Sites, New York City

Variable	Green Cart Sites^a^ (n = 265)	Candidate Sites (n = 644)	*P* Value
**Tract population, no.^b^**	5,004	3,706	<.001
**Sum population of all tracts within a quarter-mile, no.^b^**	33,529	22,963	<.001
**Tract median income, $^b^**	35,568	41,619	<.001
**Mean household income of all tracts within a quarter-mile, $^b^**	49,625	51,397	<.001
**Percentage of tract population below federal poverty level^b^**	30.1	25.6	<.001
**Proportion of tract by race/ethnicity, %^b^**			
White or Caucasian	10.9	11.7	<.001
Black or African American	30.5	49.8	<.001
Hispanic or Latino	49.1	32.5	<.001
Other	9.4	6.0	<.001
**Distance to nearest subway stop, ft^c^**	793	3,079	<.001
**No. of large employers within a quarter-mile^d^**	0.457	0.085	<.001
**No. of supermarkets within a quarter-mile^e^**	1.6	0.81	<.001
**No. other stores carrying fruits and vegetables within a quarter-mile^e^**	2.09	0.84	<.001
**No. of large employers within a half-mile^d^**	1.18	0.40	<.001
**No. of supermarkets within a half-mile^e^**	4.72	2.86	<.001
**No. of other stores carrying fruits and vegetables within a half-mile^e^**	5.4	3.1	<.001

a New York City Department of Health and Mental Hygiene list, updated periodically during 2008–2013.

b American Community Survey, 2008–2012 averages (http://factfinder2.census.gov/faces/nav/jsf/pages/searchresults.xhtml?refresh=t).

c Metropolitan Transportation Authority geographic information systems database of subway stops, 2013 (http://web.mta.info/developers/sbwy_entrance.html).

d ArcInfo Business Analyst, 2012 (Esri).

e ArcInfo Business Analyst, 2012; entries verified by using Google Street View images taken during 2007–2013.

Green Carts were positioned in and near tracts with larger population sizes, tended to be much closer to subway stops, and were more likely to be near large employers (Table 1). Most (79%) Carts were within a quarter-mile of a subway stop, compared with 28% of the candidate locations. Twenty-four (4%) candidate sites were both within a quarter-mile of a subway stop and in a food desert, representing likely economically viable yet underserved areas.

Table 2 shows the effect of neighborhood factors on the odds of Green Cart presence, comparing food deserts with areas that have at least 1 healthy food store nearby. Accounting for demographic and other neighborhood factors, Carts were found more often in areas with existing healthy food stores than in food deserts but had similar odds of being in food swamps and healthier areas. Carts had nearly 60 times the odds of being next to a subway stop compared with more than a quarter-mile away (*P* < .001), up to 3 times the odds of being in a more population-dense area (*P* < .01), and 6 times the odds of being near a large employer (*P* < .001) (Table 2, model 3, C statistic = .901). The proportion of the population living below the federal poverty level did not significantly correlate with Green Cart presence in either direction, although it was significant in univariate analyses. In model 4, higher store density was associated with up to 14 times the odds of Cart presence (C statistic =.909; *P* < .001).

**Table 2:** Odds Ratios of Neighborhood Characteristics Associated With Green Cart Placement, New York City

Variable	Model 1 (95% CI)	Model 2 (95% CI)	Model 3 (95% CI)	Model 4 (95% CI)
**Percentage of healthy food stores^a^**				
0 (Food desert)	1 [Reference]	1 [Reference]	1 [Reference]	—
0.1–20.0 (Food swamp)	6.9 (4.1–11.6)	4.6 (2.6–8.1)	2.5 (1.2–4.9)	
20.01–100 (Healthy)	6.9 (4.2–11.5)	4.2 (2.5–7.3)	2.4 (1.3–4.6)	
**Tract population^b^**				
<3,000	—	1 [Reference]	1 [Reference]	1 [Reference]
3,000–6,000		1.9 (1.3–2.9)	1.9 (1.1–3.2)	1.7 (1.0–2.9)
>6,000		2.7 (1.6–4.5)	3.2 (1.7–6.0)	2.9 (1.5–5.7)
**Percentage of tract below federal poverty level^b^**				
<10	—	1 [Reference]	1 [Reference]	1 [Reference]
10–20		1.1 (0.5–2.3)	0.6 (0.2–1.5)	0.5 (0.2–1.3)
20–30		1.2 (0.6–2.6)	1.1 (0.4–2.8)	0.7 (0.3–2.0)
≥30		1.1 (0.5–2.4)	1.2 (0.5–3.2)	0.8 (0.3–2.1)
**Percentage of black residents in tract^b^**				
<30	—	1 [Reference]	1 [Reference]	1 [Reference]
30–59.9		0.4 (0.3–0.6)	0.4 (0.2–0.6)	0.3 (0.2–0.6)
≥60		0.2 (0.1–0.4)	0.2 (0.1–0.5)	0.2 (0.1–0.4)
**Percentage of Latino residents in tract^b^**				
<30	—	1 [Reference]	1 [Reference]	1 [Reference]
30–59.9		1.2 (0.7–2.1)	1.3 (0.7–2.4)	1.1 (0.5–2.0)
≥60		1.0 (0.6–1.8)	0.5 (0.3–1.1)	0.4 (0.2–0.7)
**Distance to subway^c^**				
>a Quarter-mile	—	—	1 [Reference]	1 [Reference]
500 ft To a quarter-mile			4.2 (2.6–6.5)	3.6 (2.2–5.6)
<500 ft			59.3 (30.7–114.4)	47.5 (24.3–92.8)
**No. of large employers within a quarter-mile^d^**				
None	—	—	1 [Reference]	1 [Reference]
≥1			6.3 (3.7–10.8)	6.4 (3.7–11.3)
**No. of food stores within a quarter-mile^a^**				
0	—	—	—	1 [Reference]
1–5				2.4 (0.6–8.8)
6–15				6.7 (1.9–23.8)
>15				14.1 (3.8–52.3)

Abbreviation: CI, confidence interval; —, not included in the model.

a ArcInfo Business Analyst, 2012 (Esri); entries verified by using Google Street View images taken during 2007–2013.

b American Community Survey, 2008–2012 averages (http://factfinder2.census.gov/faces/nav/jsf/pages/searchresults.xhtml?refresh=t).

c Metropolitan Transportation Authority geographic information systems database of subway stops, 2013 (http://web.mta.info/developers/sbwy_entrance.html).

d ArcInfo Business Analyst, 2012.

Census tracts with outlier Green Carts had higher median income than those within Green Cart boundaries ($60,885 vs $33,275, *P* < .001) but were similar in other respects. Carts that accepted EBT were in poorer tracts (median income $29,850 vs $36,674 for census tracts with non-EBT Green Carts, *P* < .001). Other characteristics were no different between EBT and non-EBT Carts, including population size (*P* = .92), distance to the nearest subway stop (*P* = .67), the number of large employers (*P* = .40), or chance of being in a food desert (*P* = .46). The difference in the proportion of the population living below the federal poverty level was not significant, although it was slightly higher in tracts with Carts accepting EBT (32.6% vs 29.1% living below the poverty level, *P* = .08).

## Discussion

We found that Green Carts were rarely in food deserts and instead tended to locate near more commercial, populated areas with more pedestrian traffic. These findings are similar to those from another study on Green Cart locations in the Bronx, which, although it did not examine the food environment, found that Carts are clustered in high-traffic areas (9). This choice of location may be reasonable from an economic perspective, as population centers and areas with more traffic (near subway stops, other stores, and large businesses) increase the chances of a small business’s success (18). One report of 142 Green Cart operators in NYC found that most chose their location on the basis of the volume of foot traffic (19). However, the more highly trafficked areas where Cart vendors situate are usually near existing brick-and-mortar establishments selling healthy foods and are therefore in neighborhoods that did not necessarily need Green Carts.

We also found that many food deserts lack Green Carts, particularly those in the Bronx and Queens. We examined where Carts could have been located in addition to where they did locate to evaluate whether Green Carts are optimally placed to serve the neediest areas within Cart boundaries. Our results show that, given the limited number of available Green Cart permits, Carts could be more effectively deployed to help food desert residents, particularly those with limited mobility (eg, elderly, disabled), improve their access to fresh fruits and vegetables. Lower commercial activity, based on proximity to subway stops, large employers, and other stores, may make these food desert locations less desirable from a vendor’s perspective. When comparing findings from regression models 3 and 4, it appears that the total number of stores nearby may better predict Cart presence than the balance of healthy and unhealthy stores, as we expected; Green Carts had similar odds of being in a food swamp as in a healthier area but were significantly more likely to be in areas with more stores overall. A market-driven imperative to locate near potential customers may be in tension with the program objective of increasing access to fruits and vegetables in food deserts. Carts’ proximity to large employers and subways may also indicate that they serve a clientele beyond the produce-insufficient, low-income target population for which the program was established. This proximity may be necessary for economic viability, but further research should explore whether they also serve populations at need. Finally, many candidate sites within food deserts were identified near highly trafficked areas, and these may be economically viable sites that simultaneously improve the reach of the Green Carts program.

We expected that Green Carts accepting EBT would locate in areas with individuals more likely to receive food assistance. However, Carts that accepted EBT were no more likely to be in food deserts than Carts that did not accept EBT. This finding suggests that requiring Green Carts to accept EBT (as required in a similar pilot program [20]) may not influence more vendors to locate in higher need areas, though it may make food more affordable.

This study had limitations. First, the list of Green Carts maintained by the department of health may not be accurate, because it is not updated routinely and because Green Carts may move within the allowed boundaries of their boroughs. However, surveys of Cart vendors suggest that they do not tend to move once established (K.Y.L, E.K.C., A.M.F., C.R.H., unpublished data, 2013); therefore, our list is likely representative of where Carts typically locate. In addition, nearly half of issued Green Cart permits were not listed. These Carts may not be in operation, may only open intermittently, or may be in a location not identified by the NYC DOHMH. Our data were not temporally synchronous, although there was substantial overlap. This fact could affect study validity if there were substantial changes in the local food environment since 2012, perhaps even in response to the presence of Green Carts, as some research from the health department has suggested (21). Finally, we were unable to obtain current, census-tract level fruit and vegetable intake or body mass index (BMI) data, so we cannot draw associations between produce intake, BMI, and the food environment we have captured, though a similar relationship has been demonstrated previously (22).

The presence of Carts even in non–food desert areas may increase overall fruit and vegetable accessibility by lowering prices through competition and increasing visibility of fresh produce. The magnitude of these benefits deserves further study. Other issues not explored by our study include the seasonal nature of Green Carts, the quality and variety of produce offered, and economic viability of Carts in differing neighborhoods.

Our study demonstrated that food deserts exist in NYC and that Green Carts, which were intended to increase accessibility to fresh produce in many such neighborhoods, primarily go where people and businesses congregate, not necessarily where food deserts are. As other US cities consider similar programs to reduce disparities in access to healthy foods, it is important to assess the ways existing programs like Green Carts can be improved and the other benefits Carts may provide. Further research to determine who patronizes mobile produce vendors to see whether the vendors are reaching the target population and to explore strategies to incentivize carts to locate in food deserts may be useful.
